# Adenomatous Colorectal Polyps in Patients Referred for Colonoscopy in a Regional Hospital in Kuwait

**DOI:** 10.4103/1319-3767.65194

**Published:** 2010-07

**Authors:** Saleh A. Al-Enezi, Saqer A. Alsurayei, Ali E. Ismail, Nasser Yehia A. Aly, Waleed A. Ismail, Amany A. Abou-Bakr

**Affiliations:** 1 Department of Medicine, Farwaniya Hospital, Ministry of Health, Kuwait; 2 Department of Medicine, Faculty of Medicine, Zagazig University, Zagazig, Egypt; 3 Department of Infection Control, Farwaniya Hospital, Ministry of Health, Kuwait; 4 Department of Tropical Medicine and Hygiene, Faculty of Medicine, University of Alexandria, Alexandria, Egypt; 5 Department of Pathology, Farwaniya Hospital, Ministry of Health, Kuwait; 6 Department of Pathology, National Cancer Institute, Cairo University, Cairo, Egypt

**Keywords:** Colonoscopy, colorectal polyps, Kuwait

## Abstract

**Background/Aim:**

Adenomatous colorectal polyps (ACPs) are known to be the precursor lesions for colorectal cancer. The aim of the study was to determine the prevalence, endoscopic and pathological features of ACPs in patients referred for colonoscopy.

**Patients and Methods:**

The endoscopic and histological reports of adult patients who underwent complete colonoscopy in the gastroenterology unit of a regional Kuwaiti hospital between January 2008 and December 2008 were retrospectively studied. The specimens of polyps were reviewed by an experienced pathologist who was blinded to the clinical or endoscopic information. Non-neoplastic polyps were not included in the analysis.

**Results:**

Of 530 eligible patients (mean age, 45 years; male-female ratio, 2:1), 54 (10%) had 103 ACPs. Of the patients with ACPs (mean age, 57 years), 43 (80%) were males and 36 (67%) were Kuwaitis. Histopathological examination of the most significant polyp in each patient revealed that 40 (74%) polyps were tubular adenomas (TAs); 11 (20%), tubulovillous (TV) adenomas; and 3 (6%), villous adenomas. High-grade dysplasia was noticed in 4 (10%) adenomas. Fifteen (2.8%) of the 530 patients had advanced ACPs. Logistic regression analysis of some variables and their association with ACPs found that age (*P*<0.001; OR, 1.9; CI, 1.5-2.3), history of adenoma (*P*=0.001; OR, 6.4; CI, .2.1-19.4) and being Kuwaitis (*P*=0.029; OR, 2.1; CI, 1.1-4.1) to be independently associated with ACPs.

**Conclusion:**

The most common histological type of ACPs was tubular adenoma. Advancing age, being Kuwaiti nationals and prior removal of ACPs were significantly associated with the occurrence of ACPs.

Adenomatous colorectal polyps (ACPs) are common and known to be the precursor lesions for colorectal cancer (CRC).[1] Removal of ACPs at colonoscopic screening significantly reduces the risk of CRC.[2] Nowadays, many countries are not only adopting national CRC screening programs but are also seeking strategies to increase participation of eligible individuals in these programs.[3] It is believed that a one-time screening colonoscopy at the age of 55 years could achieve a 30 - 50% reduction in mortality from CRC.[4] Of note, the incidence of CRC has been declining in the United States due to widespread CRC screening and change in behavioral risk factors.[5]

Despite Asian countries, including Kuwait, having lower rates of CRC as compared with Western countries, studies have shown that adoption of Western lifestyles, including dietary patterns, has led to a rapid transition towards Western rates;[6,7] for that reason, screening for CRC has been enhanced as a national health priority in most Asian countries.[8]

The characteristics of both patients and polyps can predict the risk of CRC developing in an adenoma.[9] Meanwhile, the frequency and features of ACPs vary widely among different populations.[10] Understanding the natural occurrence and the features of ACPs is crucial in any CRC prevention strategy. To date, limited data on this issue is available from Kuwait and the need for a national colonoscopy screening program has not yet been assessed. The aim of the study was to determine the prevalence, endoscopic and histological features of ACPs in patients referred for colonoscopy in a regional hospital in Kuwait.

## PATIENTS AND METHODS

Endoscopic and histological reports of all adult patients who underwent complete colonoscopy up to the cecum (96% of all colonoscopies) using an Olympus colonoscope system (CF-Q 160 AL, Tokyo, Japan) in the Gastroenterology Unit of Farwaniya Hospital, Kuwait (a 920-bed regional hospital), between January 2008 and December 2008 were retrospectively studied. Pertinent clinical data were extracted from the patients’ medical records. Variables like age, gender, nationality, indication for colonoscopy, past history of ACPs and the location, morphology and histological features of ACPs were retrieved and analyzed. Patients with incomplete colonoscopy due to any cause; and those with personal or family history of hereditary polyposis syndromes, CRC or inflammatory bowel disease were excluded. A total of 530 eligible patients were enrolled.

### Study procedures

Procedures for performing colonoscopy and histological evaluation have been described elsewhere.[11] Colonoscopies were performed by 4 qualified gastroenterologists. Once the location and size of all visible polyps were identified, the polyps were removed and standard histological assessment was done in the local pathology lab. The location of ACPs was categorized as proximal or distal. Polyps at or distal to the splenic flexure were classified as distal polyps. The size of the polyp was estimated with the use of an open-biopsy forceps. Specimens were reviewed by an experienced pathologist (A.A.) who was blinded to any clinical or endoscopic information of the patients. Advanced adenoma was defined as an adenoma that was ≥10 mm and/ or was a villous adenoma (at least 25% villous) or an adenoma with high-grade dysplasia. Patients with a pathologic interpretation of intramucosal carcinoma or carcinoma *in situ* were classified in the high-grade dysplasia group. Cancer was defined as invasion of malignant cells beyond the muscularis mucosa. Patients were classified based on the most advanced lesion.[12]

### Ethical consideration

The Standing Committee for Coordination of Health and Medical Research at the Ministry of Health, Kuwait, approved this study.

### Statistical analysis

Statistical package for the social sciences (SPSS) for Windows (Version 16.0; SPSS Inc., Chicago, IL, USA) was used for analysis of data. Categorical variables were expressed as numbers and percentages. Chi-square or Fisher’s exact test, where appropriate, was used for analysis of categorical variables. Continuous variables were expressed as medians, or as means and standard deviation, as appropriate. Differences in means were compared using Student *t* test. Significant potential risk factors for CRC in univariate analysis were tested in a logistic regression model. All tests used were two-tailed. A *P* value <0.05 was considered statistically significant.

## RESULTS

### Demographic characteristics of the cohort of eligible patients

Of the 530 eligible patients (mean age, 44.8±14.3 years), 54 (10%) had 103 ACPs. Additional 13 (2.5%) patients had had non-neoplastic polyps and were not considered in the analysis. Two hundred sixty-seven (50%) patients were Kuwaitis (K): 187 were male (M), 80 were female (F) and 263 (50%) were of other nationalities (178, M; 85, F). The overall M-F ratio was about 2:1. There were no significant age or gender differences between Kuwaitis and non-Kuwaitis (NK) (*P*>0.05). ACPs were detected in 36 Kuwaiti patients (28M, 8F; 13% of Kuwaiti patients) and in 18 non-Kuwaiti patients (13M, 5F; 7% of non-Kuwaiti patients). The overall prevalence of ACPs among all males in the cohort irrespective of nationality was 11% (41/365); and among all females, 8% (13/165). The distribution of age, gender, nationality and occurrence of ACPs among all patients is shown in Table 1.

**Table 1:** Distribution of patients according to age, gender and nationality

Variable	Nationality			
	Kuwaiti		Non-Kuwaiti	
Age, years (mean±SD)	(46.3 ±15.7)		(43.3 ±12.5)	
**Gender**	**No. (%)**	**No. (%) with polyps**	**No. (%)**	**No. (%) with polyps**
Male	187 (35)	28 (5)	178 (34)	13 (2)
Female	80 (15)	8 (2)	85 (16)	5 (1)
Total	267 (50)	36 (7)	263 (50)	18 (3)

The site distribution of 103 ACPs among the affected 54 patients revealed that 62 were in distal colonic site and 41 in proximal colonic site. Concerning patients, 31 had 53 isolated distal polyps, 7 had concurrent 9 distal and 7 proximal polyps while 16 had 34 isolated proximal polyps. Table 2 shows site distribution, sizes and shapes of the 103 ACPs.

**Table 2:** Distribution and morphology of 103 adenomatous colorectal polyps

Variable	
Distribution of ACPs (no., %)	
Distal colorectal position	62 (60)
Proximal colonic position	41 (40)
Shape (no., %)	
Sessile	65 (63)
Pedunculated	38 (37)
Size (no., %)	
1-5 mm	61 (59)
6-9 mm	31 (30)
10 mm or more	11 (11)
Size of polyps in mm, median (range)	5 (2-25)
Number of polyps/patient, median (range)	1 (1-12)

### Demographic characteristics of patients with ACPs

Of the 54 patients with ACPs, 43 (80%) were males and 36 (67%) were Kuwaitis. Their mean age (56.6 years ± 12.7) was significantly higher than that of those without ACPs (*P*=0.001). Past history of adenoma was reported in 8 (15%) patients. The most common indications for colonoscopy among those patients were abdominal pain, constipation and bleeding per rectum (39%). Table 3 summarizes the demographic characteristics and indications for referral in patients with ACPs.

**Table 3:** Demographic characteristics of, and indications for referral in, patients with adenomatous colorectal polyps

Variable	
Age in years, mean ± SD	56.6 ± 12.7
Gender, male (no., %)	43 (80)
Nationality, Kuwaiti (no., %)	36 (67)
Past history of adenoma (no., %)	8 (15)
Indications for referral (no., %)	
Abdominal pain	21 (39)
Bleeding per rectum	21 (39)
Constipation	21 (39)
Post-polypectomy surveillance	16 (30)
Weight loss	8 (15)
Anemia	8 (15)
Bloating	6 (11)
Diarrhea	6 (11)

### Size and histopathological types of the most significant ACPs

In general, the size of the polyp ranged between 2 and 25 mm (median, 5 mm). A polyp size ≥10 mm was identified in 11 (20%) patients. Results of histopathological examination of the most significant polyp in each patient are shown in Table 4.

**Table 4:** Size and histopathological types of the most significant adenomatous colorectal polyps

Type	Tubular adenoma (TA)	Tubulo-villous adenoma (TVA)	Villous adenoma (VA)
No. patients with (%)	40 (74)	11 * (20)	3 *(6)
Size of ACP			
≥10 mm	1*	7†	3†
<10 mm	39	4	-

* advanced polyps;

† 2/7 patients with TVA and 2/3 patients with VA had highgrade dysplasia

### Advanced ACPs

Fifteen patients (2.8% of the entire cohort of patients) had advanced ACPs. There was no significant difference between patients with advanced ACPs and those without regarding nationality, gender, past history of adenoma, site distribution or indication for colonoscopy (*P*>0.05). However, a polyp size of ≥10 mm and patient age ≥40 years were significantly associated with advanced polyp pathology (*P*< 0.001 and 0.006, respectively).

### Distribution of patients with ACPs in groups according to age

The overall prevalence of the most significant and advanced ACPs increased significantly with age. The majority of patients with ACPs (41/54, 75.9%) had ages ≥50 years [Table 5].

**Table 5:** Distribution of patients with the most significant adenomatous colorectal polyps according to their age groups

Age group	All patients	Patients with advanced polyps No. (%)	Patients with non-advanced polyps No. (%)	All patients with polyps No. (%)
<40 years	213	1 (0.5)	4 (1.8)	5 (2.3)
40-49 years	101	4 (4.0)	4 (4.0)	8 (7.9)
50-59 years	123	5 (4.1)	13 (10.5)	18 (14.6)*
≥60 years	93	5 (5.4)	18 (19.3)	23 (24.7)*
Total	530	15 (2.8)	39 (7.4)	54 (10.2)

* Refers to patients with ACPs who had age ≥50 years (total 41 patients)

### Variables and their association with ACPs

Categorical data analysis showed that ACPs were significantly associated with Kuwaiti nationals (*P*=0.005) and past history of adenoma (*P*<0.001). Logistic regression analysis of some variables and their association with ACPs is shown in Table 6. Age (*P*< 0.001; OR, 1.9; CI, 1.5-2.3), past history of adenoma (*P*=0.001; OR, 6.4; CI, .2.1-19.4) and being Kuwaiti nationals (*P*=0.029; OR, 2.1; CI, 1.1-4.1) were independently associated with ACPs.

**Table 6:** Logistic regression analysis of some variables and their association with adenomatous colorectal polyps

	*P*	Adjusted OR	95.0% CI of OR	
			Lower	Upper
Kuwaiti nationals	0.029	2.105	1.081	4.099
Past history of adenoma	0.001	6.426	2.120	19.480
Age/10 *	<0.001	1.860	1.484	2.332
Gender	0.161	1.683	.813	3.484
Constant	0.000	0.010	-	-

* Age for each 10-year increment

## DISCUSSION

This report is the first to address the profile of ACPs in a cohort of symptomatic adult patients in Kuwait. Despite being a non-population-based study, significant data are presented. In our patients, the prevalence of ACPs was substantial among patients aged 50 years or more, and a considerable number of these patients had advanced adenoma.

The prevalence rates of ACPs vary considerably. An earlier study from Kuwait[13] reported a prevalence of 4.6% for various types of colorectal polyps. In our study, the overall prevalence was comparatively higher. This might be attributed to higher mean age of our patients. Studies from different Asian countries on symptomatic patients showed a prevalence of 5.1% in India,[10] 11.7% in Iran[14] and 14.8% in 10 Asian countries according to reports from 17 endoscopy centers.[15]

The variability in prevalence may be due to dissimilarity in indications for colonoscopy and in the proportion of patients who had high risk for ACPs, such as men, older patients, patients with positive fecal occult blood test and those with family history of CRC.[16–19]

As reported in other studies,[14,20] we did not find any significant association between the referring indication for colonoscopy and the detection of ACPs. However, in contrast to the notion that male gender predicts higher prevalence of adenoma,[21–23] we found no significant gender-related association. This may be attributed to the relatively smaller number of females in this study. However, Bafandeh *et al*. similarly found no association between gender and occurrence of ACPs in symptomatic Iranians referred for colonoscopy.[14]

A significant number of ACPs were proximally located. Advanced proximal neoplasia may be missed if screening is done only by sigmoidoscopy.[11,24,25] In our study, proximal ACPs were detected more frequently in patients with distal ACPs than in those without distal ACPs, which is supported by a meta-analysis of screening colonoscopy studies.[26]

In addition, no age or gender difference with regard to site distribution of overall and advanced ACPs was observed in our patients; this has been noted in other studies as well.[10,27] However, Imperiale *et al*.[24] and Lieberman *et al*.[12] have observed that age is an important risk factor for all proximal neoplasias regardless of distal findings. In addition, Anderson *et al*.[28] found male gender to be predictive of non-advanced proximal neoplasia.

Twenty-six percent of our patients with ACPs had villous component. In other studies, villous adenomas ranged from 10% to 38%.[10,29] Villous polyps may become malignant in 29% to 70% of the cases.[30]

The prevalence of advanced ACPs was slightly higher than that reported by Cheng *et al*.[18] (1.3%) but much lower than that reported by Sung *et al*.[17] (12.5%); and that reported by Lam *et al*. (33%),[31] who reported a peculiarly increasing prevalence of advanced colonic polyps in young patients undergoing colonoscopy in Hong Kong. Advanced ACPs have been reported to develop into cancer and to be predictive, after removal, of future advanced neoplasia.[32] In our study, large ACPs were more likely to demonstrate advanced pathology. However, carcinoma *in situ* and invasive cancers are sometimes found in small tubular adenomas (TA).[33]

Increased polyp size, villous histology and severe dysplasia are all associated with an increased risk of malignancy in an adenoma. This risk is even greater for those who have multiple polyps.[34] A total of 14 (26%) of our patients with ACPs had two or more polyps and thus carried high-risk adenomas. Additionally, and similar to other studies,[1,35–37] it was found that the prevalence of overall and advanced adenomas increases substantially with advancing age, being more common over the age of 50 years.

Our study revealed a strong association between past history of colorectal adenomas and the adenoma detection on surveillance colonoscopy. Past history of adenoma increases the incidence of recurrent overall and advanced neoplasia.[28,32] In addition, Kuwaiti nationals had almost 2-fold increased risk of ACPs when compared to non-Kuwaitis. The reasons for this higher risk are not clear. It is probable that higher exposure to western-type fatty diet and consequently higher incidence of obesity in Kuwaitis[38] may be a contributory factor.

Although Kuwait is considered to be among the countries with low incidence of colorectal cancer, there has been an increasing trend in age-standardized rate (ASR) of colorectal cancer among Kuwaitis since 1988.[39]

The last web-published data from the International Agency for Research on Cancer regarding cancer incidence in Kuwait showed ASR (per 100,000) during the period 1998-2002 for the colon and rectum cancers to be 8.4 and 5.2, respectively.[40] Local unpublished data from the cancer registry in Kuwait has even shown further rise in ASR (per 100,000) of colon cancer to 9.2 among males and 9.5 among females.

It is relevant to mention that there is no screening program for CRC in Kuwait so far. Recommending a screening program requires population-based studies in asymptomatic average-risk individuals (aged ≥50 years), and its implementation will require strong collaboration between the primary health care sector and the specialized endoscopy centers.

Our study has certain limitations in that certain risk factors for ACPs like body mass index, smoking, alcohol and dietary pattern were not studied because of the restrictions associated with the retrospective nature of the study.

In conclusion, the prevalence of overall and advanced ACPs in our patients was significant. Most of the significant polyps were identified in patients aged 50 years or older, had a tubular type in histology and a considerable proportion of them had advanced pathology. Advancing age, being Kuwaiti nationals and past history of ACPs were significantly associated with the occurrence of ACPs.
